# Are component positioning and prosthesis size associated with hip resurfacing failure?

**DOI:** 10.1186/1471-2474-11-227

**Published:** 2010-10-02

**Authors:** David R Marker, Michael G Zywiel, Aaron J Johnson, Thorsten M Seyler, Michael A Mont

**Affiliations:** 1 Center for Joint Preservation and Replacement, Rubin Institute for Advanced Orthopedics, Sinai Hospital of Baltimore, 2401 West Belvedere Avenue, Baltimore, MD USA; 2 Department of Orthopaedic Surgery, Wake Forest University Health Sciences, Winston-Salem, North Carolina USA

## Abstract

**Background:**

Recent studies suggest that there is a learning curve for metal-on-metal hip resurfacing. The purpose of this study was to assess whether implant positioning changed with surgeon experience and whether positioning and component sizing were associated with implant longevity.

**Methods:**

We evaluated the first 361 consecutive hip resurfacings performed by a single surgeon, which had a mean follow-up of 59 months (range, 28 to 87 months). Pre and post-operative radiographs were assessed to determine the inclination of the acetabular component, as well as the sagittal and coronal femoral stem-neck angles. Changes in the precision of component placement were determined by assessing changes in the standard deviation of each measurement using variance ratio and linear regression analysis. Additionally, the cup and stem-shaft angles as well as component sizes were compared between the 31 hips that failed over the follow-up period and the surviving components to assess for any differences that might have been associated with an increased risk for failure.

**Results:**

Surgeon experience was correlated with improved precision of the antero-posterior and lateral positioning of the femoral component. However, femoral and acetabular radiographic implant positioning angles were not different between the surviving hips and failures. The failures had smaller mean femoral component diameters as compared to the non-failure group (44 versus 47 millimeters).

**Conclusions:**

These results suggest that there may be differences in implant positioning in early versus late learning curve procedures, but that in the absence of recognized risk factors such as intra-operative notching of the femoral neck and cup inclination in excess of 50 degrees, component positioning does not appear to be associated with failure. Nevertheless, surgeons should exercise caution in operating patients with small femoral necks, especially when they are early in the learning curve.

## Background

With recent studies reporting early clinical success in greater than 90% of metal-on-metal total hip resurfacing arthroplasty patients, there has been renewed interest in this modality as a bone-conserving alternative to standard total hip arthroplasty [1-3]. However, surgeons adopting resurfacing should be aware that these results are reflective of institutions where the orthopaedic surgeons have extensive experience and have performed a large number of these procedures [4-7]. Hip resurfacing arthroplasty is a technically difficult procedure that has a corresponding long learning curve. Early in this curve, there may be increased risk for complications that require revision surgery such as femoral neck fracture, femoral component failure, and component loosening [8,9].

Recent studies have elucidated many patient-related as well as surgical and implant-related factors that may increase the risk for these complications [4,9,10]. Some patient-specific factors, such as neuromuscular or neurosensory deficiency that might adversely affect gait or weight-bearing, and documented allergy to cobalt, chromium, or molybdenum, are generally accepted as absolute contraindications. Other patient characteristics are considered relative risk factors. For example, presence of multiple cysts, females of childbearing age, and osteopenia are often interpreted as relative contraindications by experienced surgeons. In addition to these patient-related variables, surgical factors such as femoral neck notching have been identified as increasing the risk for failure. Other recent studies have suggested that component positioning and implant size may be associated with risk for dislocation and increased levels of metal ions [11-17]. In general, it is recommended that the femoral component be placed in a valgus position in order to reduce tension and shear stresses. Other studies have suggested that the preferred stem-shaft angle be less than 140 degrees to avoid notching [11,16,18]. Cup inclination between 30 degrees and 50 degrees with version between 5 degrees and 15 degrees are recommended by some authors for maximum range of motion and avoidance of impingement [17,19].

Early in the learning curve for a surgeon, there is the potential for more variability in prosthetic positioning. Some authors have reported cadaveric studies with the use of computer navigation to investigate this relationship, [20-22] but to the knowledge of the authors, there are no clinical studies that have assessed whether this correlation between surgeon experience and implant positioning exists.

The purpose of this study was to assess a prospective cohort of patients at a single institution to evaluate the role of surgeon experience on implant positioning and the subsequent association with implant survival. More specifically, we asked two primary questions: 1) Does implant positioning change with surgeon experience?; and 2) does this positioning and component size have an association with implant longevity?

## Methods

We evaluated the first 361 consecutive metal-on-metal total hip resurfacing arthroplasties (257 in men and 104 in women) performed by a single surgeon that had a minimum follow-up of 28 months (mean of 59 months, range 28 to 87 months). At the time of operation, the mean patient age was 50 years (range, 18 to 79 years), and the mean body mass index was 27 kg/m^2 ^(range, 16 to 48 kg/m^2^). The preoperative diagnoses were osteoarthritis for 269 hips, post-traumatic arthritis for fourteen hips, dysplasia for thirteen hips, osteonecrosis for fifty-six hips, and inflammatory arthritis for nine hips. Institutional review board approval was obtained for this study, and no informed consent was required from patients to be included in this review.

The patients were screened as acceptable candidates for hip resurfacing based on standard criteria. All patients underwent standard pre-operative physical evaluation and review of their medical history to determine if they were candidates for surgical intervention. If patients were not skeletally mature or at least 18 years old, they were excluded from the study. In addition, any patient with a positive human chorionic gonadotropin test or evidence of active human immunodeficiency virus (HIV) or hepatitis infection was not considered for this procedure. Neuromuscular or neurosensory deficiency that might adversely affect gait or weight bearing and documented metal allergies were considered absolute contraindications.

All procedures were performed by the senior author using an anterolateral approach. The components had a metal-on-metal bearing from the same manufacturer (Conserve Plus; Wright Medical Technology, Arlington, Tennessee). The acetabular component was placed without screws using cementless press fit fixation, and the femoral component was cemented in all cases. A standard rehabilitation protocol was utilized; patients were recommended to increase weightbearing over a ten week period. They started with 20% weightbearing restrictions using two crutches for the first six weeks, 50% restrictions with a cane or a crutch in the opposite hand between six and ten weeks, and full weightbearing as tolerated thereafter.

Standard antero-posterior radiographs of the pelvis and the operated hip, as well as direct lateral radiographs of the operated hip, were obtained for all patients in the recovery room following the index arthroplasty. A radiographic assessment of cup inclination on antero-posterior radiographs and stem-neck angles in the antero-posterior and direct lateral views were compared between the early and late groups. Acetabular inclination was determined relative to the horizontal line tangential to the inferior margin of both ischial tuberosities. Pre-operative anatomic neck-shaft angles were measured using the line that passes through both the center of the femoral head and mid-point of the isthmus of the femoral neck (neck axis), as well as the line that passes through the center of the femoral canal at points 5 and 10 cm distal to the lesser trochanter (shaft axis). The stem-shaft angles on post-operative radiographs were measured using the line that passes through the center of the base and tip of the femoral component stem (stem axis), and the previously-described shaft axis. The difference between the stem-shaft angle and the anatomic neck-shaft angle was considered to be the stem-neck angle.

Based on a recent study from the present authors' center describing a markedly higher rate of mechanical complications in the first 100 metal-on-metal resurfacings, the patients were stratified into two groups: early in the learning curve (first 100 procedures) and late in the curve (the remaining 261 surgeries) [9]. At approximately the hundredth case that the surgeon made modifications to the patient selection and surgical technique, based on the high complication rate in the early cases. In summary, these modifications included avoiding resurfacing in patients with large femoral head or neck cysts, ensuring proper seating of the femoral component, and ensuring an optimal thickness of the cement mantle.

The precision of the component placement was assessed by determining the standard deviation of the antero-posterior and lateral stem-shaft angles, as well as the cup inclination angles for consecutive groups of 50 patients on a rolling basis (for example, the standard deviation in angles for patients 1 through 50, patients 2 through 51, and so forth).

The patients were also grouped by whether or not they required component-based revision surgery (received a revision by the time of final follow-up or were scheduled for a revision procedure). There were 31 patients who required revision. Two patients who were revised for acetabular complications in the immediate post-operative period (one loose cup that were revised the same day, and one loose cup revised for an intra-operative acetabular fracture) were excluded from the failure analysis. Additionally, four patients who were revised because of a peri-prosthetic infection, and two patients with soft-tissue complications (one abductor mechanism rupture, and one persistently painful hip non-responsive to non-operative treatment with extensive scar formation seen intra-operatively at the time of revision surgery) were also excluded from the failure analysis. Of the remaining 23 patients, the reasons for revision were femoral neck fracture (n = 13), acetabular cup loosening (n = 2), femoral component loosening (n = 4); femoral component fracture (n = 2), and late acetabular protrusion (n = 2). As previously reported, 15 of these complications occurred within the first 50 cases, whereas the remainder were distributed approximately equally among the remaining 311 cases [9]. The radiographic measurements of cup and stem-shaft angles as well as component sizes of the failures and non-failures were compared to assess for any differences that might have been associated with an increased risk for failure.

Data was prospectively collected in a database for the overall cohort of resurfacing patients. The data were subjected to analysis using SigmaStat version 3.0 software (Systat Inc, San Jose California) and MedCalc version 10.2 software (Medcalc software, Mariakerke Belgium). A Student's t-test was used to calculate the probability that there were differences in the radiographic measurements between the early and late learning curve cohorts. Variance ratio testing was utilized to compare the standard deviations of the early and late cohorts. Linear regression analysis was used to evaluate whether there was a correlation between the precision of component placement and the number of cases performed by the surgeon. The failure and non-failure radiographic measurements and implant sizes were compared using a Student's t-test. Assessment of the correlation between the radiographic values and Harris Hip scores was made using Pearson's coefficient with a linear analysis. All statistical comparisons were conducted using 95% confidence intervals where a p value of less than 0.05 was considered significant.

## Results

No significant difference was found in the antero-posterior and lateral stem shaft angles over time, while a small but significant difference was seen in the cup inclination angles. The mean antero-posterior stem shaft angle for the early cohort was 2 degrees valgus (range, 20 degrees varus to 28 degrees valgus; SD = 7 degrees), and the late cohort had a mean stem-neck angle of 1 degrees of valgus (range, 24 degrees varus to 25 degrees valgus; SD = 5 degrees) (p = 0.107). Similarly, the lateral stem-neck angles were similar for the two groups with means of 2 degrees of retroversion (range, 18 degrees of anteversion to 18 degrees of retroversion) versus 3 degrees of retroversion (range, 19 degrees of anteversion to 25 degrees of retroversion) relative to the femoral neck (p = 0.091). There was a significant difference (p = 0.036) in cup inclination, with a mean of 35 degrees (range, 14 to 54 degrees) in the early group versus 37 degrees (range, 18 to 65 degrees) in the hips that were performed later in the learning curve. A full description of findings can be found in Table 1.

**Table 1:** Comparison of early and late groups

				correlation between precision and experience	
	Early group (n = 100)	Late group (n = 261)	p-value	coefficient	p value
Mean anteroposterior stem-shaft angle in degrees (range)	2 (-20 to 28)	1 (-24 to 25)	0.107	0.64	< 0.001
Standard deviation of anteroposterior stem shaft angle in degrees	7	5	0.012		
Mean lateral stem-shaft angle in degrees (range)	2 (-18 to 18)	3 (-19 to 25)	0.091	0.37	< 0.001
Standard deviation of lateral stem shaft angle in degrees	7	6	0.595		
Mean acetabular cup inclination angle in degrees (range)	35 (14 to 54)	37 (18 to 65)	0.036	0.12	0.027
Standard deviation of acetabular cup inclination angle in degrees	8	7	0.275		

NB: negative values indicate varus positioning in the anteroposterior plane, and anteversion in the lateral plane

**Figure 1:**
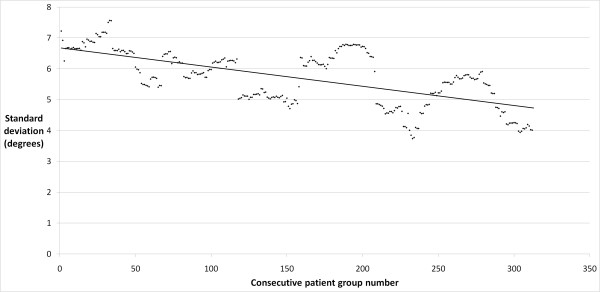
**Plot of antero-posterior stem-shaft angle standard deviation in consecutive rolling 50 patient groups**.

**Figure 2:**
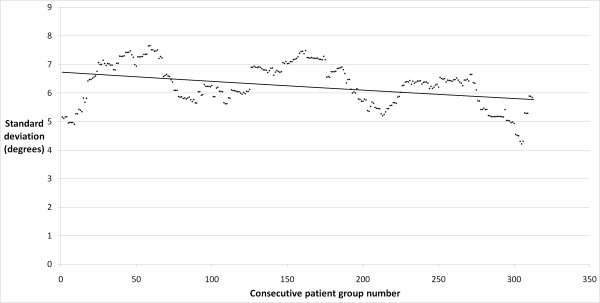
**Plot of lateral stem-shaft angle standard deviation in consecutive rolling 50 patient groups**.

**Figure 3:**
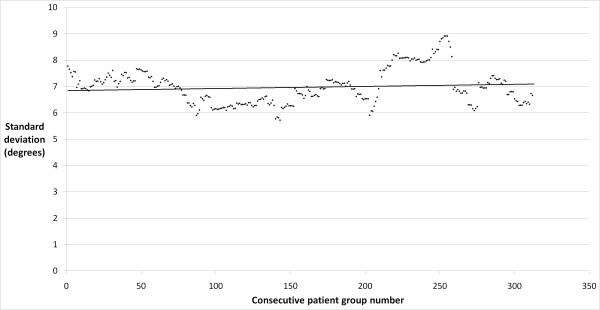
**Plot of acetabular cup inclination angle standard deviation in consecutive rolling 50 patient groups**.

The precision of the placement of the femoral component in the coronal plane improved significantly with experience, with a decrease in the antero-posterior stem-neck angle standard deviation from 7 degrees to 5 degrees in the early and late cohorts, respectively (p = 0.012). There was a strong correlation between the number of cases performed by the operating surgeon and the precision of component placement (r = 0.64; p < 0.0001; Figure 1). The precision of the placement of the femoral component in the sagittal plane was similar between the early and late groups, with standard deviations of 7 and 6 degrees, respectively (p = 0.595). A moderate correlation was found between surgeon experience and an improvement in the precision of component placement in this plane (r = 0.37; p < 0.001; Figure 2). The precision of the acetabular cup placement in terms of inclination angle was similar between the early and late groups, with standard deviations of 8 and 7 degrees, respectively (p = 0.275). A weak and clinically insignificant inverse correlation was found between the surgeon's experience level and the precision of acetabular cup placement (r = 0.13; p = 0.027; Figure 3).

No significant differences were observed in any of the radiographic angles evaluated between the failures and surviving hips (Table 2), but the femoral component sizes were significantly smaller in the patients who experienced a component failure, with mean femoral component diameters of 44 millimeters (range, 36 to 54 millimeters) versus 47 millimeters (range, 36 to 56 millimeters) for the failure and non-failure groups, respectively (p < 0.001).

**Table 2:** Comparison of failure and non-failure groups

				correlation	
	Failures (n = 23)	Non-failures (n = 330)	p-value	coefficient	p value
Mean anteroposterior stem-shaft angle in degrees (range)	0 (-14 to 12)	2 (-24 to 28)	0.488	0.08	0.725
Mean lateral stem-shaft angle in degrees (range)	1 (-18 to 19)	3 (-19 to 25)	0.219	0.27	0.283
Mean acetabular cup inclination angle in degrees (range)	37 (20 to 60)	37 (14 to 60)	0.875	0.17	0.428

NB: negative values indicate varus positioning in the anteroposterior plane, and anteversion in the lateral plane

## Discussion

The learning curve associated with hip resurfacing remains a repeatedly discussed topic in the scientific literature. Although relative inexperience with this surgical technique has been identified as a factor contributing to complications such as gross femoral neck notching, the association between this learning curve and implant positioning remains largely undefined. This question served as one of the reasons for conducting the present study as we assessed whether implant positioning changes with surgeon experience and whether this positioning and component sizing have an association with implant longevity?

This study has several limitations. The first is the short follow-up period. Despite the ability to collect data such as implant positioning in the immediate post-operative period, other factors evaluated (e.g. femoral neck fracture, component loosening) may not become apparent until years after the procedure. Because of this, it is important to follow this patient cohort to mid- and long-term follow-up to ensure that the conclusions regarding the correlation between implant position and complications rates remain the same.

The results of this study suggest that there was greater precision in the placement of the femoral component in the resurfacings performed later in the surgeon learning curve, and that the importance of surgeon experience is especially pronounced with regard to varus-valgus positioning. Furthermore, the precision of component placement continued to improve throughout the patient series, suggesting that surgeons may need to perform several hundred hip resurfacing procedures before a plateau is reached in the precision of placement. In order to aid in the learning process, the senior author recommends the use of intra-operative fluoroscopy to ensure adequate acetabular component seating and to minimize the possibility of intra-operative acetabular fracture, as well as to strictly follow the manufacturer's recommendations regarding the appropriate cement mantle for femoral component placement to ensure proper seating. However, the clinical importance of these findings is unclear as there was no association between implant positioning and the risk for failure, and as previously reported by the present authors, the incidence of mechanical failure requiring revision was markedly reduced following the first 50 to 100 cases. Interestingly, there was a statistically, but not clinically significant inverse correlation between surgeon experience and the cup inclination angles. It should be emphasized that the r-value of the correlation is extremely small, indicating that there is a large variance in the data around the least-squares correlation model. Despite these results, in showing a slightly lower precision of acetabular component placement, it is also important to emphasize that these findings should be interpreted as resulting in no clinical change in the component placement as the acetabular component was still placed within the recommended parameters.

In contradistinction to these findings, recent studies have reported that positioning is correlated with outcome. Beaule et al. reported that hips with a stem-shaft angle of less than or equal to 130 degrees had an increase in the relative risk of an adverse outcome by a factor of 6.1 (p < 0.004) [11]. Other factors besides implant positioning that should be considered concerning implant survival are complete coverage and full seating of the femoral component, as well as maximizing its size. These techniques have been shown to reduce overall complication rates from over 13% to approximately 2% and femoral neck fracture rates from 7.2% to 0.8% [23]. In addition, there may be a role for navigation systems that can be used in the early part of the surgeon learning curve to improve implant positioning [24].

A number of studies have also begun to assess the correlation of implant positioning and the level of metal ions. Hart et al. reported that there is a threshold level of 50 degrees for cup inclination. They found significant differences in whole blood cobalt (p < 0.01) and chromium (p = 0.01) levels in patients who were below versus above this threshold [25]. The mean blood cobalt and chromium below this threshold were 1.6 ppb and 1.88 ppb, and above this threshold, they were 4.45 ppb and 4.3 ppb. Similarly, Langton et al. reported that metal ion concentrations in patients who had smaller femoral components (less than or equal to 51 millimeters) were significantly related to the inclination (p = 0.01) and anteversion (p = 0.01) of the acetabular component [13]. While the present study did not measure metal ion levels, the results did not indicate an association with implant failure and acetabular inclination. However, it is possible that because of the low number of high acetabular cup inclination angles as well as acetabular failures, the present study was insufficiently powered to detect such an association. In addition, these results reflect short to mid-term follow-up and longer-term assessments are necessary to more fully analyze any detrimental affect due to implant positioning and the role of metal ion levels for the risk of implant failure or other possible adverse events.

Similar to the results of the present study, several other recent reports have suggested that larger prosthesis size may be associated with more favorable outcomes. Smith et al. assessed metal-on-metal implants of varying size using a hip joint simulator [26]. Dynamic motion and loading cycles that simulated walking for both lubrication and wear studies demonstrated that the steady-state wear rate for 36 millimeter diameter prostheses (0.07 cubic millimeters per 10^6 ^cycles) was lower than for smaller prosthetic sizes. They suggested that the surface separation they found during considerable parts of each walking cycle was evidence of the formation of a protective lubricating film. In a multicenter clinical study, Stulberg et al. reported similar findings in that thirteen (17%) of the seventy-eight patients with a 40 or 44-mm femoral component had a failure compared to eleven (4.2%) of 259 patients with a 48, 52, or 56-mm component (p = 0.001) [8]. While implant size is largely determined by patient body habitus and skeletal dimensions, these findings suggest that surgeons might choose the larger sizing if there are multiple viable options, and they are not sacrificing acetabular bone.

## Conclusions

The results of the present study suggest that surgeon experience improves the precision of component placement. However, in the absence of recognized risk factors such as intra-operative notching of the femoral neck and cup inclination angles in excess of 50 degrees, component placement, in and of itself, was not associated with an increased risk of failure. The present study did not find any clear association with positioning and implant longevity. Surgeons should exercise caution in operating patients with small femoral necks, especially when they are early in the learning curve, as larger component size was associated with fewer failures. Additional studies are needed to further assess the clinical importance of increased metal ions due to implant positioning.

## Competing interests

No financial support was received directly in support of this study, although some of the data was collected as part of an IDE study funded by Wright Medical Technologies. MAM is a consultant for Stryker Orthopaedics and Wright Medical Technologies, receives royalties from Stryker Orthopaedics, and receives research and/or institutional support from Stryker Orthopaedics, Wright Medical Technologies, Tissue Gene, and the National Institutes of Health (NICHD & NIAMS). None of the other authors have any competing interests to disclose.

## Authors' contributions

DRM, MGZ, AJJ, TMS, MAM designed the study. DRM, MGZ, TMS collected the data. DRM, MGZ, AJJ, TMS analyzed the data. DRM, MGZ, MAM prepared the manuscript., MGZ, AJJ, MAM ensured the accuracy of the data and analysis. All authors have read and approved the final manuscript.

## Pre-publication history

The pre-publication history for this paper can be accessed here:

http://www.biomedcentral.com/1471-2474/11/227/prepub
